# Supplementary material for: Self healable neuromorphic memtransistor elements for decentralized sensory signal processing in robotics
Source: Nat Commun. 2020 Aug 12;11:4030. doi: 10.1038/s41467-020-17870-6 (PMC7424569; doi:10.1038/s41467-020-17870-6)
Supplement: Supplementary file 1 — Supplementary Information [file 41467_2020_17870_MOESM1_ESM.pdf]

<u>Supporting Information</u>

Self Healable Neuromorphic Memtransistor Elements for Decentralized Sensory Signal Processing in Robotics

John et al.

Supplementary Note 1. Device structure and DC electrical behaviour of Satellite Threshold Adjusting Receptors (STARs)

To build a STAR, we adopt a standard thin film transistor architecture with indium-tungsten oxide (IWO) serving as the semiconducting channel and an ionic liquid 1-ethyl-3-methylimidazolium bis(trifluoromethylsulfonyl) imide, [EMI][TFSI] serving as the ionic dielectric (Supplementary Figure 1a). Hosting the ionic liquid in a poly (vinylidene fluoride-co -hexafluoropropylene) P(VDF-HFP) housing allows convenient fabrication of free-standing films and lamination on to the desired substrate *via* the 'cut and stick' processing strategy[1]. The high dielectric constant of the ionic liquid enables application of these films as ionic dielectrics in our thin film transistor configuration. To quantify the dielectric characteristics (Supplementary Figure 1b), specific capacitance of a 10 μm thick P(VDF-HFP) ion gel (measured by a scanning electron microscope (SEM) cross-sectional image (Supplementary Figure 1c)) is measured as a function of frequency ($C = -1/2\pi fZ''$, where Z'' is the imaginary impedance and f is frequency) using a dielectric spectrometer. The measured capacitance depicts a decreasing trend with frequency with the highest value of ~7.5 μFcm^{-2} reported at low frequency (1 Hz). This large value results from the formation of electrical double layers at the electrode/ion gel interface. For transistor mobility calculations, the specific capacitance value of 7.5 μF cm^{-2} is used in accordance with literature[1].

Supplementary Figures 1d-e shows the typical I–V curves of the threshold switching behaviour of our devices from -1.5 to +1.5 V with a sweeping rate of 0.05 Vs^{-1}. The devices switch from their off to on-state at a threshold voltage (V_{th}) of ~-0.2 V. On removal of the input voltage, the device returns to its off-state spontaneously at a voltage around ~-1 V, depicting a typical threshold switching behaviour. The top-gated ionic-mode transistors exhibit a field-effect mobility of 79 cm^{2}V^{-1}s^{-1} operating at ultralow voltage (V_{gs}=1.5 V and V_{ds}=10 mV) and power. The linear field-effect mobility is extracted from the transfer characteristics using the equation $\mu = (dI_d/dV_g) \times (L/WC_iV_d)$, where L is the channel length (300 μm), W is the channel width (1000 μm) and C_i is the gate dielectric capacitance. The large electrical-double-layer (EDL) capacitance results in efficient carrier accumulation within the small electrochemical window of the ion-gels, resulting in ultralow voltage and power operation. The devices also exhibit a large anticlockwise hysteresis window of 0.8 V. This is attributed to migration-relaxation kinetics of mobile ions in the dielectric, which in turn accumulates and dissipates carriers for the channel formation based on the direction of voltage scan[2]. Forward scan results in migration

of cations towards the ion-gel – semiconductor interface, accumulating electrons in the IWO layer to form a conductive channel. This results in lower voltage requirements for the subsequent channel formation in the reverse scan, accounting for the anticlockwise hysteresis. Supplementary Figure 1f shows the statistics of the on-off ratio of 20 FETs measured in the same V_{gs} widow (-1.5 to +1.5 V, sweeping rate= 0.05 Vs^{-1}) at a constant V_{ds}= 0.1 V.

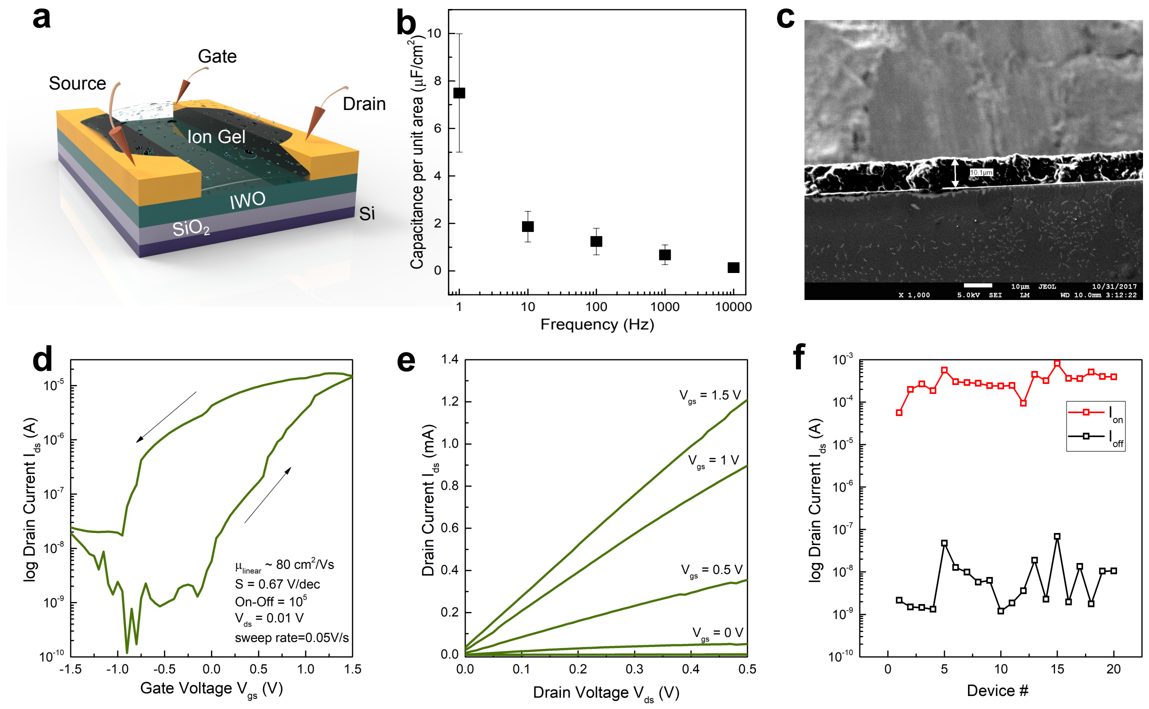

Supplementary Figure 1. Transistor Characteristics. **a** Device structure. Satellite Threshold Adjusting Receptors (STARs) are realized in an electric-double-layer transistor configuration with semiconducting IWO channels and solid-state ionic dielectrics based on P(VDF-HFP)-[EMI][TFSI]. To demonstrate the unique features of our STARs, voltage triggers representing external stimuli are input at the gate terminal and the corresponding output current responses are recorded as a function of time. **b** Frequency dependence of specific capacitance for the free-standing ion gel dielectric. **c** shows scanning electron microscopy image of the ion gel. **d** Transfer and **e** Output characteristics of the diffusive transistors/STARs. **f Statistics of the field-effect transistor (FET) performance.** Distribution of the on-off ratio of 20 FETs measured in the same V_{gs} widow (-1.5 to +1.5 V, sweeping rate= 0.05 Vs^{-1}) at a constant V_{ds}= 0.1 V.

Supplementary Note 2. Normal-state operation, Relaxation and Working Principle of STARs

Normal-State Response

In their normal state, defined here as the current response of the device when subjected to pulses of V_{gs}=1 V prior to application of any injury pulses ($V_{gs} \geq 2$ V), the STAR's response is unable to reach the pain threshold (I_{nox}=3.3 mA) even when stimulated by an extensive train of

voltage triggers (number~ 50). Increasing the amplitude of voltage triggers enhances the current response and reduces the incubation time.

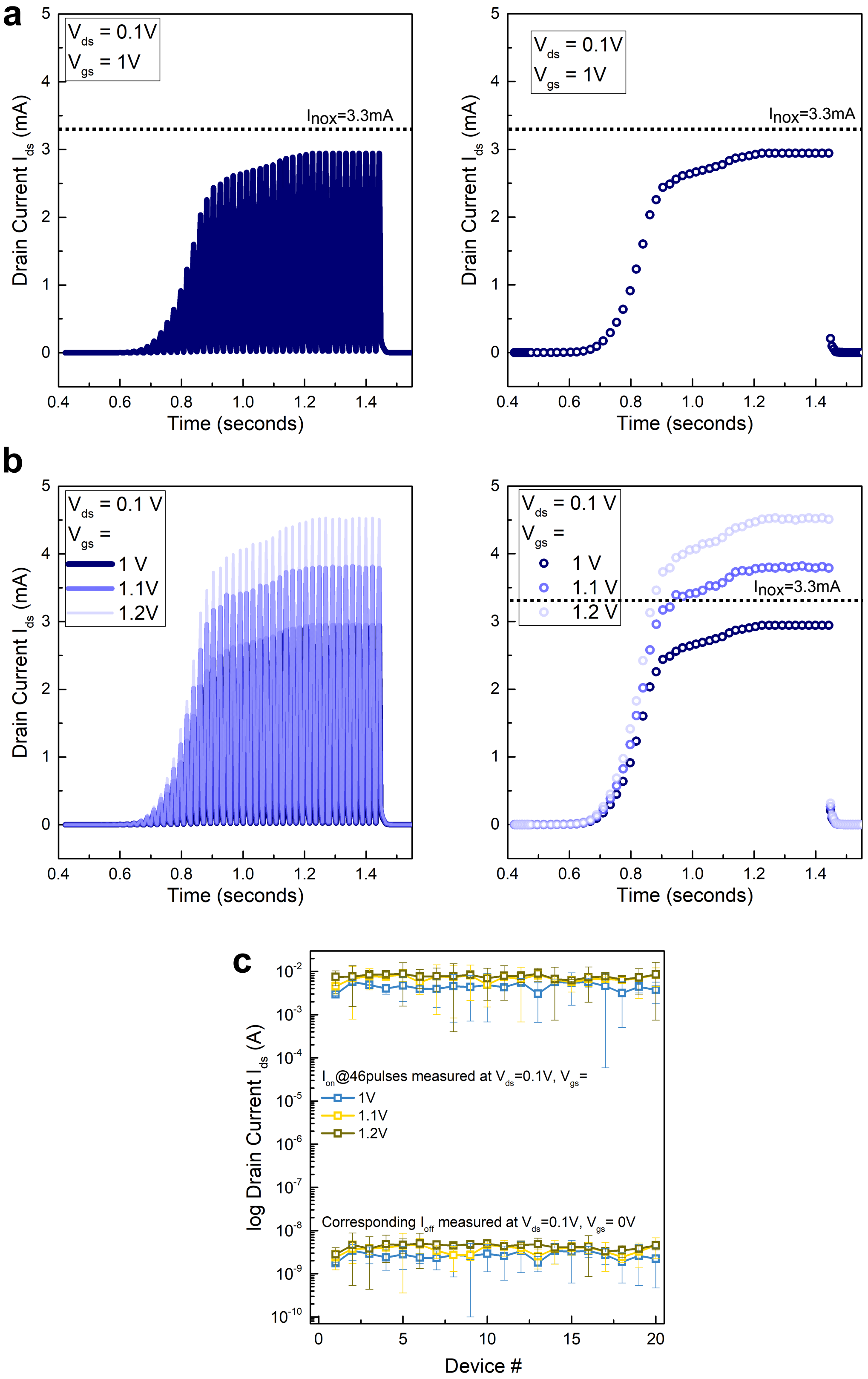

Supplementary Figure 2. Satellite Threshold Adjusting Receptors (STARs). a Normal-state operation of STARs. In this work, the normal-state operation of our STARs is defined as the current response of the device when subjected to pulses of V_{gs}= 1 V, prior to application of any injury pulses ($V_{gs} \geq$ 2 V). Left: shows the raw output current spikes, Right: shows the peak points of current response. **b** Response of the device to multiple number of 16ms pulses with different amplitudes (V_{gs}= 1, 1.1, and 1.2 V). Higher amplitudes result in shorter incubation times (1.18, 0.88, and 0.86 ms, respectively) and higher output currents. **c Switching endurance of STARs.** Switching endurance of 20 devices measured across 100 cycles each. Each cycle consists of 46 voltage pulses applied at the gate terminal at a constant V_{ds}= 0.1 V. The cycle-to-cycle variations are captured by the error plots, while the graph itself shows the variation between devices. As can be seen, since the switching is volatile, the switching endurance is very good and remains stable for the duration tested.

Supplementary Note 2 Supplementary Figure 2 shows the output current response to trains of 16 ms triggers of different amplitudes (1.0–1.2 V). Since the output response of STARs go beyond the pain threshold I_{nox}=3.3 mA with continuous pulsing of V_{gs}=1.1 V and 1.2 V, these could be considered as noxious stimuli. The incubation time (time taken to reach the pain threshold I_{nox}= 3.3 mA) depict an inverse relation with the amplitude of the voltage triggers, indicating the dosage-dependent threshold-switching behaviour in our devices. For example, on increasing the amplitude of voltage triggers to 1.1 V, the pain threshold could be attained in 23 pulses and the current response reaches a maximum of 3.79 mA at the end of the pulse train. On the other hand with 1.2 V pulses, the pain threshold could be attained in 20 pulses and the current response reaches a maximum of 4.51 mA at the end of the pulse train.

Relaxation Behaviour

Relaxation in nociceptors is defined as a phenomenon where innocuous triggers immediately following warning or noxious stimuli could trigger warning responses from devices due to the short-term memory effects induced by a priori warning stimuli[3]. This is done to cater to the often repeated nature of such stimuli; i.e., innocuous triggers immediately following noxious stimuli are also considered noxious for a particular interval to overprotect the injured site. To demonstrate the phenomenon of relaxation, paired pulse measurements are carried out with different interval times (5-32 ms) and a response of $I_{relaxation}$= 21 nA is set as the threshold for relaxation/warning as shown in Supplementary Figure 3. Single voltage spikes that generate a response higher than $I_{relaxation}$=21 nA are referred to as warning stimuli (1.1-1.2 V) and those that generate a response lower than $I_{relaxation}$ are called innocuous triggers (0.5-1 V).

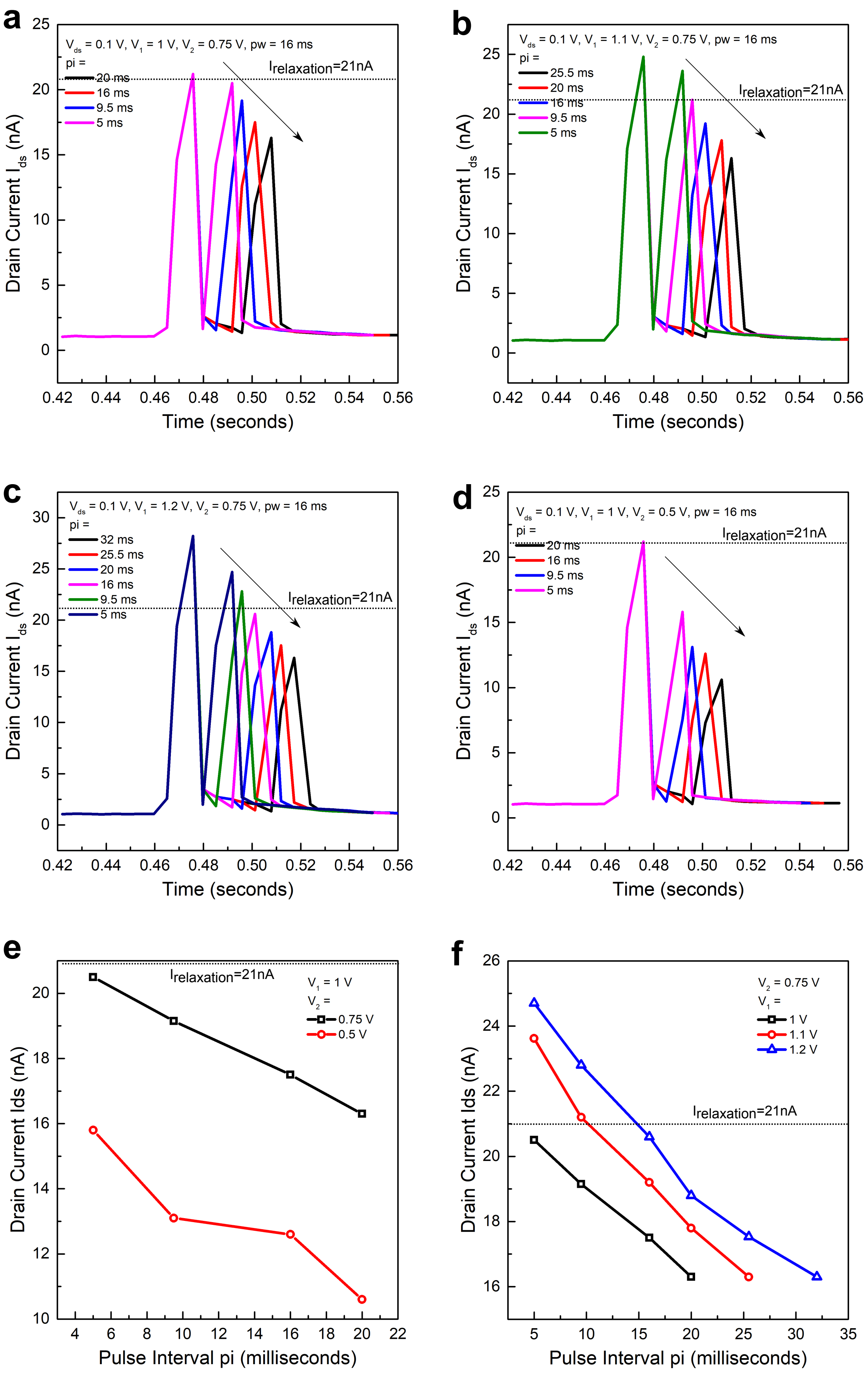

a
Vds = 0.1 V, V1 = 1 V, V2 = 0.75 V, pw = 16 ms
pi =
20 ms
16 ms
9.5 ms
5 ms
Irelaxation=21nA
Drain Current Ids (nA)
Time (seconds)
b
Vds = 0.1 V, V1 = 1.1 V, V2 = 0.75 V, pw = 16 ms
pi =
25.5 ms
20 ms
16 ms
9.5 ms
5 ms
Irelaxation=21nA
Drain Current Ids (nA)
Time (seconds)
c
Vds = 0.1 V, V1 = 1.2 V, V2 = 0.75 V, pw = 16 ms
pi =
32 ms
25.5 ms
20 ms
16 ms
9.5 ms
5 ms
Irelaxation=21nA
Drain Current Ids (nA)
Time (seconds)
d
Vds = 0.1 V, V1 = 1 V, V2 = 0.5 V, pw = 16 ms
pi =
20 ms
16 ms
9.5 ms
5 ms
Irelaxation=21nA
Drain Current Ids (nA)
Time (seconds)
e
Irelaxation=21nA
V1 = 1 V
V2 =
0.75 V
0.5 V
Drain Current Ids (nA)
Pulse Interval pi (milliseconds)
f
V2 = 0.75 V
V1 =
1 V
1.1 V
1.2 V
Irelaxation=21nA
Drain Current Ids (nA)
Pulse Interval pi (milliseconds)

Supplementary Figure 3. Relaxation behaviour of Satellite Threshold Adjusting Receptors (STARs). On removal of the noxious stimuli, the STARs enter the relaxation phase during which it recalibrates/increases its sensitivity to cater to the often repeated nature of such stimuli; i.e., innocuous triggers immediately following noxious stimuli enhances the nociceptive response. **a-d** shows the raw output currents of the relaxation characterization using priori pulses (V_1) followed by successive innocuous pulses (V_2) with varying interval times (5-32 ms). The current responses follow an inverse trend with pulse interval, reminiscent of the enhanced sensitivity of the nociceptor within the relaxation process; and could be further tuned as a function of the amplitudes of the triggers. **e-f** depicts a summarized view of this trend.

Here, we basically demonstrate that innocuous triggers can generate significant response (>21 nA for paired pulse measurements) if they follow closely priori triggers that generate a strong warning response. For example, innocuous triggers (0.75 V) following priori triggers (1.1 V) that arrived after the device fully relaxed (> 5 ms) did not change the device state. However when the pulse intervals are kept short ($\leq$5 ms), significant responses ($\geq$21 nA) are triggered in our devices, depicting the relaxation behaviour (Supplementary Figure 3b). This interval window could be further tuned by modulating the amplitude of the priori and successive triggers as shown in Supplementary Figure 3c. For example, larger amplitude of the priori triggers (1.2 V) extends the relaxation interval (interval in which innocuous triggers are identified as noxious) to 9.5 ms. On the other hand, on arrival of extremely weak successive stimuli (0.5 V), the devices remain unperturbed even in their relaxation phase (Supplementary Figures 3a, d, e), reiterating the volatile threshold switching property of our devices. Supplementary Figures 3e-f summarizes the relaxation behaviour. When both the priori and subsequent triggers are innocuous, the warning threshold $I_{relaxation}$= 21 nA is never reached (Supplementary Figure 3e), but when the innocuous triggers closely follow noxious triggers, the warning threshold $I_{relaxation}$= 21 nA is easily crossed and the relaxation time can be further modulated by the amplitude and pulse interval of the priori and following spikes (Supplementary Figure 3f). In short, this short-term memory although immediate could be utilized to establish temporal correlations between the noxious stimuli and hence, contributes to the learning process.

Working Principle

Configured as gated-threshold switches, our STARs functionally emulate the signal processing of a biological nociceptor. Application of positive voltage pulses to the gate terminal drives the imidazolium cations in the ion gel dielectric toward the dielectric–semiconductor interface,

which in turn causes accumulation of electrons in IWO layer to form a conductive channel. Upon removal of the voltage pulse, the cations gradually drift back to their equilibrium positions, which reduces channel conductivity and the drain current gradually decreases back to the resting current, resulting in the large anticlockwise hysteresis window seen in Supplementary Note 1, Supplementary Figure 1c. The accumulation of electrons the IWO layer during the forward scan results in lower voltage requirements for the subsequent channel formation in the reverse scan, accounting for the anticlockwise hysteresis.

Electrical triggers mimicking the external stimuli induce output responses from the STARs above V_{on}~ -0.2 V (Supplementary Note 1, Supplementary Figure 1c). A high current pulse at the output corresponds to the perception of pain in response to noxious stimuli. The STAR subsequently relaxes back to its off-state upon removal of the stimulus and gets ready to respond to the next stimulus. Gated via the ionic gel, this spontaneous relaxation back to its initial state or volatility in switching is caused by the back diffusion or relaxation of ions in the dielectric upon removal of the field stimulus, in turn decreasing the channel conductivity. This ion migration-relaxation dynamics at the semiconductor– dielectric interface defines the volatile short-term memory/plasticity behaviour in our devices and is harnessed to present the temporal dynamics of artificial nociceptors or STARs. This is functionally analogous to the working of conventional 2-terminal diffusive memristors and hence, we refer to this as the diffusive mode of STARs/ gated-threshold switches.

Weak electrical triggers ($<V_{on}$) do not cause switching in STARs and the devices stay at its off-state with no/negligible output current, suggesting the innocuous nature of the stimuli. The I-V relationship allows us to set flexible thresholds for pain in the devices. Intense noxious stimuli ($>>V_{on}$) causes temporary resistive-switching in STARs, temporarily reducing the V_{on} for channel formation and increasing the output current response as explained below. From the device perspective, this temporary resistive switching in STARs is caused by the onset of generation of additional oxygen vacancies in the semiconducting channel upon persistent stressing of the ionic interface[4]. However, the ion gel being semi-permeable allows intercalation of oxygen back into the semiconducting layer upon removal of the voltage stimuli, enabling passive functional healing of the injured devices. The same functional healing process can also be actively cured by applying pulses of opposite polarity, intercalating oxygen back into the semiconducting channel on demand.

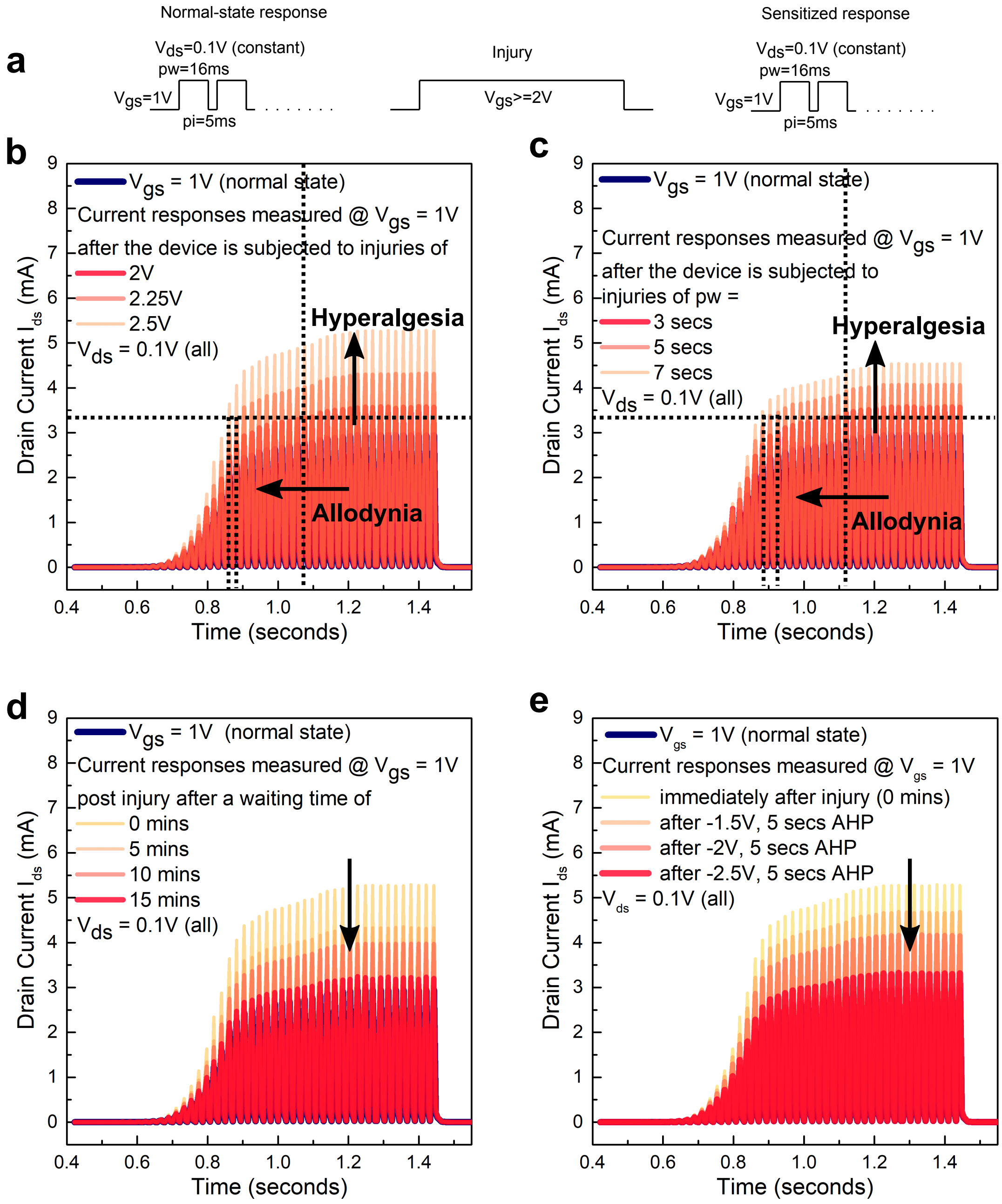

Supplementary Figure 4. Nociceptive Signal Processing in Satellite Threshold Adjusting Receptors (STARs). Pulsed response of STAR. Response of the device to a train of input voltage pulses of width 16ms and amplitude 1 V. Raw output current response corresponding to Fig. 2 (main text). **a** As indicated by the legends, the normal-state responses are initially measured at a V_{gs}= 1 V. Noxious stimuli or Injury pulses ($V_{gs}\geq$2 V) are next applied on the gate terminal of the STARs, after which the sensitized current response curves are once again measured at V_{gs}= 1 V (refer to the waveform on top). Increased amplitude **b** and pulse width **c** of the noxious stimuli enhance the current response (hyperalgesia) and reduce the incubation time/threshold (allodynia), akin to biological nociceptors. **b** The device is subjected to injuries represented by voltage shocks of 2 V, 2.25 V and 2.5 V, pulse width=3 secs. The sensitized responses are measured post-injury at V_{gs}= 1 V. **c** The device is subjected to injuries represented by voltage shocks of 2 V, pulse width= 3, 5 and 7 secs. Here, the sensitized responses are measured immediately after the application of noxious stimuli at V_{gs}=1 V. The threshold switching behaviour also

enables **d** passive healing with time and **e** active healing with curing pulses of opposite polarity. For **d**, the device is subjected to an injury of 2.5 V, pulse width=3 secs. The responses are measured post-injury at V_{gs}= 1 V after waiting for 5, 10 and 15 minutes respectively. For **e**, the device is subjected to an injury of 2.5 V, pulse width= 3 secs. Next, active healing pulses (AHP) of -1.5, -2 and -2.5 V are applied for 5 secs. The responses are next measured post-healing at V_{gs}=1 V. The spike waveform shown on top represents the inputs applied on the STARs to generate the normal and sensitized responses.

Sensitization

In Fig. 2 (main text) and Supplementary Figure 4, output current of 3.3 mA is set as the pain threshold in STARs. Stimuli resulting in outputs higher than 3.3 mA, i.e. $V_{gs} \geq 2$ V are considered as noxious while lower amplitude pulses represent innocuous stimuli (V_{gs}= 1 V). Intense noxious stimuli increases the firing rates of biological nociceptors as a function of its intensity to indicate severity of the injury. Similarly, our diffusive transistor enhances its current response and switches faster, proportional to the amplitude of the input electrical shocks.

Sensitization + Relaxation

The ion migration-relaxation kinetics of the transistors remain valid throughout six orders of magnitude of current of the FET operation, enabling demonstrations of short-term memory across several magnitudes of conductance. This allows us to set a very flexible threshold for pain perception in comparison to conventional 2-terminal memristors. To corroborate this point (Supplementary Figure 5), here the normal state responses are initially measured in STARs by application of a pulse train at V_{gs}= 1 V (stage i). The device is next allowed to relax for 50 ms (stage ii). The relaxation state responses are measured by the application of a pulse train at V_{gs}= 0.75 V. 0.75 V is chosen here because it is an innocuous stimulus, in line with our earlier definition (stage iii). Because the priori stimuli were not of noxious nature (V_{gs}= 1 V), the short-term memory/relaxation effect is negligible and the subsequent current response measured at V_{gs}= 0.75 V does not cross the pain threshold I_{nox}= 3.3 mA. A noxious stimulus or injury pulse (V_{gs}=2.5 V) is next applied on the gate terminal of the STAR (stage iv - not shown in the Figure). The sensitized state responses are next measured by application of a pulse train at V_{gs}= 1V. The pain threshold I_{nox}=3.3 mA is crossed as expected (stage v). The device is allowed to relax for 50 ms (stage vi) and the new relaxation state responses are measured by the application of a pulse train at V_{gs}= 0.75 V (stage vii). Now, since the device has experienced a noxious stimuli as its antecedent (V_{gs}= 2.5 V), the relaxation effect becomes very prominent and causes the current to cross the pain threshold I_{nox}=3.3 mA even when measured at an

innocuous voltage stimulus of V_{gs}= 0.75 V. This represents how the relaxation effects heavily depend on the nature of the priori stimuli and its interval.

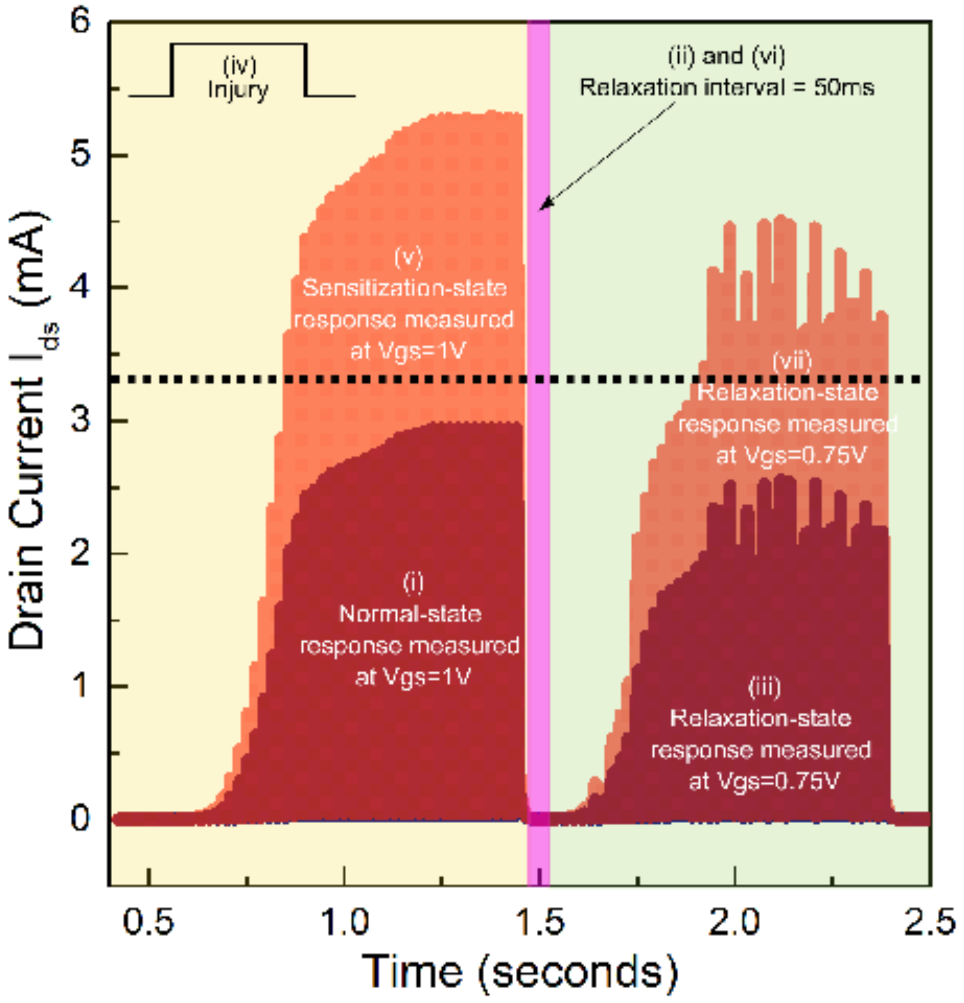

Supplementary Figure 5. Relaxation and Nociception in Satellite Threshold Adjusting Receptors (STARs). Combining relaxation and nociception, the sliding threshold function allows to filter significant noxious information from other sensory information, and fuses temporal correlations with noxious information to enable lower-order processing of sensory signals.

Supplementary Note 3. CMOS implementation of STAR

The threshold behaviour exhibited by STARs can be mimicked using conventional transistors as well but would require many more devices. Supplementary Figure 6a shows an example circuit that can exhibit all the threshold modulating properties reported earlier for STAR devices, while Supplementary Table 1 presents the values of the components as used in the

circuit. Input pulse amplitudes at V_g denote the intensity of noxious stimuli similar to Fig. 2 (main text). The NMOS transistor M2 converts that into a pulsed current that is provided as input to a PMOS current mirror comprising M1-M6, while the capacitor C sets a time constant for memory. M3-M4 is an inverter that inverts the polarity of the input pulse at V_g to turn on M5 and thus enable output current only when there is an input pulse. With multiple input pulses, the voltage on node V_{g6} would slowly reduce which would result in an increase in output current from M6. With an increased magnitude of the input pulse at V_g, this charging would happen faster and hence, a threshold will be reached quicker similar to the phenomenon of hyperalgesia and allodynia. This circuit was simulated using 65 nm CMOS transistor models with a power supply voltage of V_{dd} = 1.2 V. The results are shown in Supplementary Figure 6b for 3 different magnitudes of the input pulse in the range of 0.6-0.7 V. As expected, the output current from M6 increases with the number of pulses at a rate defined by the capacitor and input pulse magnitude. For higher values of input amplitude, the rate of increase is faster mirroring the response of STARs shown earlier.

a

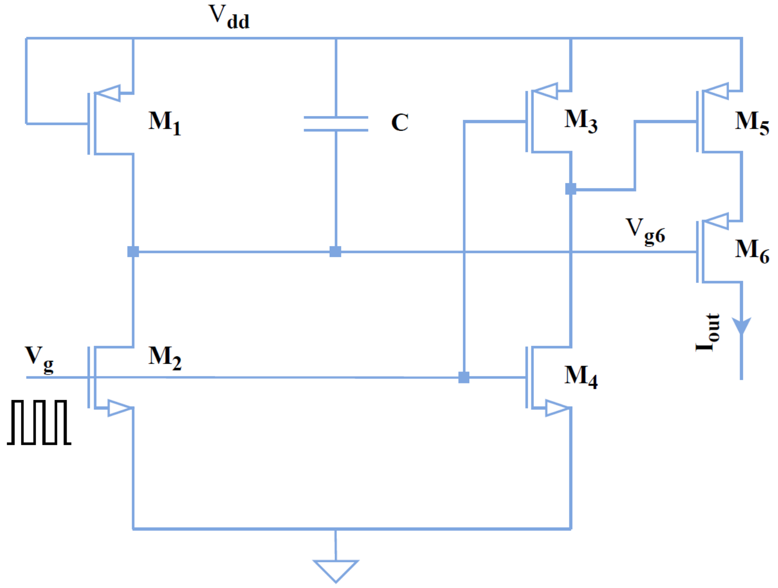

b

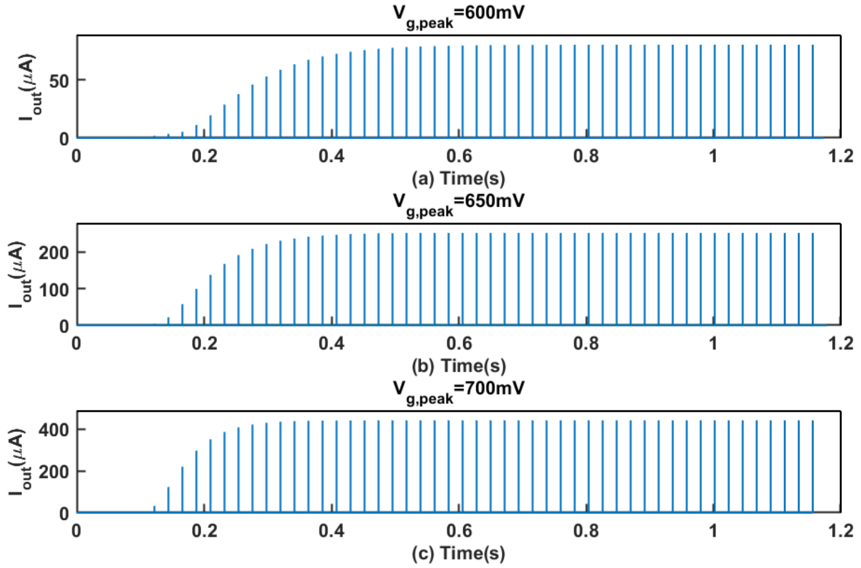

Supplementary Figure 6. CMOS implementation of a Satellite Threshold Adjusting Receptor (STAR). a Circuit diagram comprising 6 transistors and 1 capacitor. **b** Simulated output of the CMOS STAR circuit using 65nm CMOS transistor models for different magnitude of input pulses indicating different intensities of noxious stimuli (a-600 mV, b-650 mV, c-700 mV). For increased input voltage magnitude, a threshold is reached earlier, similar to Fig. 2 (main text) representing hyperalgesia and allodynia.

Supplementary Table 1. Values of various components of CMOS implementation of STAR

Component	Width (μm)	Length (μm)	Value
M_1	0.5	0.06	-
M_2	1	0.5	-
M_3	0.12	0.06	-
M_4	0.24	0.06	-
M_5	5	0.06	-
M_6	5	0.06	-
C	44.4	44.4	4pF

Supplementary Note 4. Satellite Weight Adjusting Resistive Memories (SWARMs) Working Principle and Learning Rules

Configured as gated-memristive switches, SWARMs functionally emulate the signal processing of a biological synapse. In this case, the input voltage pulses are optimized in amplitude, pulse width and frequency to induce resistive switching in our SWARMs. Operating in the top ionic gated mode, extraction and intercalation of oxygen from the ultra-thin semiconducting channels modulates the oxygen vacancy concentration in the thin film, in turn

modulating the channel conductivity permanently and enabling long-term potentiation (LTP), depression (LTD) and STDP[4].

On persistent application of voltage pulses with higher amplitude, the electrolyte-gating approach creates additional oxygen vacancies, modulating the local electronic structure of the channel, and resulting in a non-volatile memory. Application of high electric fields have been shown to trigger both electrical and structural transitions in a wide variety of materials, including insulator-metal/ Mott transitions [5–7], structural changes in VO_2[8], $MoTe_2$ [9], $SrCoO_{2.5}$ [10] and $SrTiO_3$[5,6]. The electrolyte acts as a permeable membrane for oxygen extraction/intercalation from/into the semiconducting channel. To systematically examine field-driven vacancy creation, we present the transfer curves of the FETs in the programmed and erased states (Supplementary Figure 7a). To simplify the analysis, only the forward sweeps of the transfer characteristics are taken into consideration. Constant positive biasing of the ionic top-gate at +1.5 V for 10 pulses results in a negative shift of the threshold voltage (V_{th}) and higher off (I_{off}) and on-currents (I_{on}), indicating generation of excess carriers for charge transport. Biasing with higher fields or longer durations results in a further programmed shift of V_{th}, I_{on} and I_{off} to more conductive low resistance states (LRSs), confirming this observation. Pulses of opposite polarity -1.5 V (erasing) shifts back the transfer curves to the original high resistance state (HRS). This stochiometric modulation of the channel defines the non-volatile long-term memory/plasticity behaviour in our devices and is harnessed to present the temporal dynamics of artificial synapses or SWARMs. This is functionally analogous to the working of conventional 2-terminal drift memristors and hence, we refer to this as the drift mode of SWARMs/ gated-memristive switches.

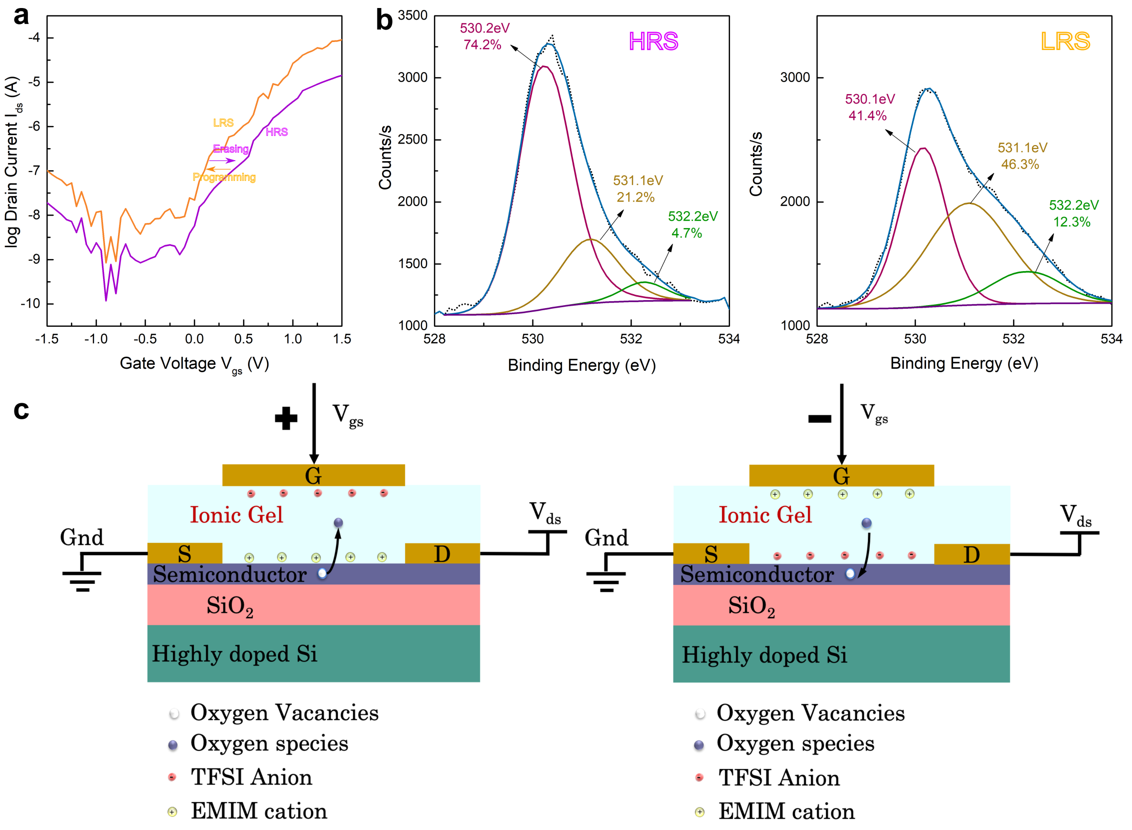

Supplementary Figure 7. Switching mechanism of Satellite Weight Adjusting Resistive Memories (SWARMs). **a** Transfer curves of the field-effect transistors (FETs) in the programmed and erased states. **b** X-ray photoelectron spectroscopy (XPS) analysis of the O 1s peak depicts an increase in the oxygen vacancy concentration in low resistance state (LRS) when compared to high resistance state HRS. **c** Schematic of the extraction and intercalation of oxygen from and into the semiconducting channel[4].

To get a more direct evidence of the underlying mechanism, X-ray photoelectron spectroscopy (XPS) measurements were performed and analysed for the metal-oxygen bonding (Supplementary Figure 7b), before and after application of voltage biasing, i.e. in their HRS and LRS. The O 1s peak was analysed to estimate the modulation of oxygen vacancies since the conduction pathways in these oxides are predominantly dictated by vacant spatially dispersed ns orbitals,. The O 1s peak was deconvoluted to three individual peaks located around 530.1, 531.1, and 532.2 eV.[11] The peak at the lowest binding energy (~530.1 eV) was assigned to the oxygen atoms in the fully oxidized indium environment (lattice oxygen; M−O−M). The mid-peak at ~531.1 eV was assigned to oxygen ions in the oxygen-deficient region (indicative of oxygen vacancy concentration). And the peak at high binding energy (~532.2 eV) was assigned to the presence of loosely bound oxygens (adsorbed oxygen) associated with the presence of hydroxyl groups on the surface.[12] Upon biasing the devices at +1 V for 1200 s, the

percentage area of M-O-M peak, oxygen vacancy peak and adsorbed oxygen peak changed from 74.2 % to 41.4 %, 21.2 % to 46.3 % and 4.7 % to 12.3 % respectively. The increase in the percentage of the oxygen-deficient region indicates an increase in the concentration of oxygen vacancies for thin films upon biasing. Films biased at lower dosages (1 V, 15 s) does not show any difference in the M-O bonding in the XPS analyses (data not shown). This aligns with the volatile switching observed in STARs. Please note that the bias voltage applied for the XPS measurements on SWARMs are much stronger than that used to map the LTP-LTD weight updates. This is intentionally done so as to ensure excellent non-volatile memory retention between the time of biasing and XPS measurements, in order to pick up differences in the oxygen vacancy concentration in the HRS and LRS.

The modulation of oxygen vacancies is evident in terms of the shift in threshold voltage and increase in on-state current in the transfer characteristics curve of ionic liquid gated thin film transistor. Based on these observations we propose that prolonged application of this electric field facilitates extraction of oxygen (creation of oxygen vacancies) from the semiconducting lattice to generate additional carriers for charge transport, resulting in a permanent shift of V_{th} and an increase in I_{on}. Higher electric fields accelerate this extrusion process, shifting the V_{th}, I_{on} and I_{off} by larger amounts. Application of electric field in the opposite direction (negative biasing) intercalates oxygen back into the semiconducting channel and shifts the electrical parameters back towards the original state (Supplementary Figure 7c).

Spike-timing-dependent plasticity (STDP)

A refinement of Hebb's theory, STDP is considered to be the first law of synaptic plasticity and forms the basis of associative learning.[13,14] The precise relative timing of pre- and postsynaptic spikes significantly affects the sign and magnitude of long-term synaptic modification. To characterize this property, the initial channel conductance (G_{ds}) is read by a reading spike (V_{read}= +0.1 V, 10 ms). In our memtransistor configuration, this reading voltage is applied across the drain and source terminals or in other words V_{read}= V_{ds}. Next, spike patterns (V_{write}) corresponding to Supplementary Figure 8 are then applied between the gate (V_g) and source (V_s) terminals and the change in conductance/weight (ΔG_{ds}) is recorded as a function of the pulse interval between pre- and postsynaptic spikes. The timing difference create effect writing voltages ($f(V_{pre}-V_{post}), t$) across the device, which on crossing the threshold voltage, creates long-term weight changes in the channel. The resultant conductance change is

finally read again with the V_{read} pulse. Weight changes are predominant at small pulse intervals, and weakens with increase in the interval, reflecting strong temporal correlations between the pre- and postsynaptic spikes. The STDP time windows shown here in milli-seconds and weight changes are comparable to biological values and could be further tuned by modulating the pulse width, number and shape of the input spikes.[15] Operating in the top ionic-gated mode, the SWARMs depict an anti-Hebbian[16] form of the STDP function where a causal temporal order of first pre-synaptic activity followed by postsynaptic activity leads to long-term depression (LTD) while the reverse order leads to long-term potentiation (LTP).

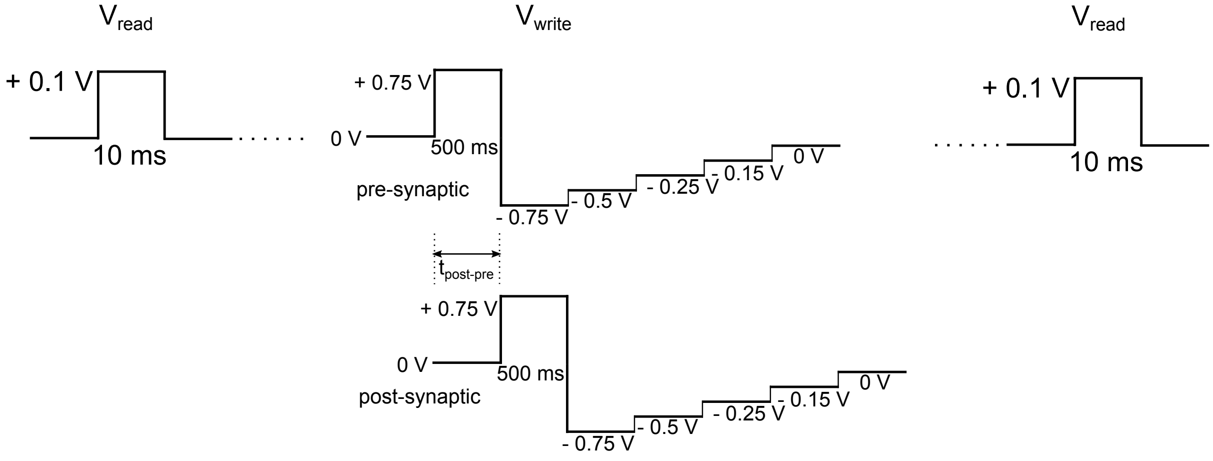

Supplementary Figure 8. STDP in Satellite Weight Adjusting Resistive Memories (SWARMs). Input waveforms used for spike-timing dependent plasticity (STDP) measurements in the top ionic-gated mode. 10 consecutive pulses were applied as input to get stable non-volatile weight changes with an average retention of ~ 20-30 minutes. A read pulse of + 0.1 V is applied at the drain terminal to read the conductance states before and after the STDP write operations.

Supplementary Note 5. CMOS Satellite Spiking Neurons (SSNs)

A CMOS satellite spiking neuron (SSN) circuit (Supplementary Figure 9) is constructed to mimic the signalling pathway of biological peripheral nervous system (PNS). The SWARM is denoted by R_{mem} and the circuitry in Supplementary Figure 9 yields an output pulse train (Q) with a pulse rate that is approximately proportional to the current through the memristive device (R_{mem}). In other words, the firing rate of spikes at Q increases due to a reduction of the memristance and vice versa, thereby emulating the behaviour of an integrate and fire (I&F) neuron that also acts as a current-to-frequency converter (converting noxious stimulus intensity to firing rate for example).

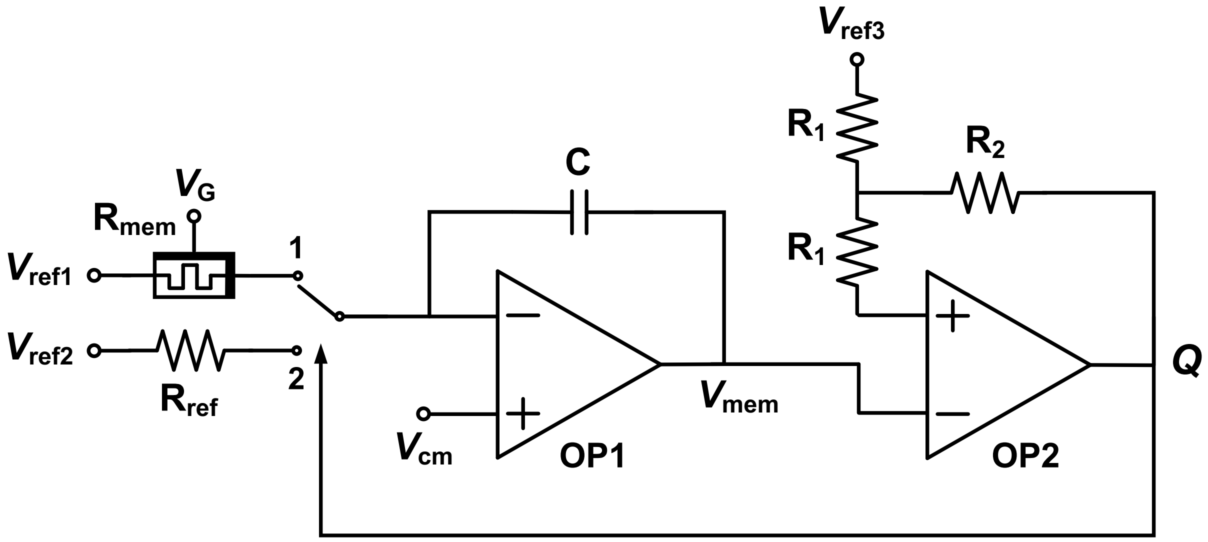

Supplementary Figure 9. Satellite Spiking Neuron (SSN) Circuit. Electronic circuit designed to mimic the behaviour of a sensory spiking neuron, where the output (*Q*) pulse rate changes in proportion to the input current through the memresistance (R_{mem}).

The circuit comprises of an active RC-integrator using operational-amplifier (op-amp) OP1 to create membrane potential V_{mem} by integrating the input current through R_{mem}. This is followed by an op-amp based Schmitt trigger (OP2) acting as a comparator with hysteresis (hysteresis window $\Delta V_o = V_{th,up} - V_{th,lo}$) that compares V_{mem} with an internally set threshold $V_{th,lo}$. The output of the Schmitt trigger (*Q*) is further used to control the charging or discharging of the integrator capacitor (C), by means of the single-pole double-throw (SPDT) switch (implemented by a 2:1 multiplexer) due to which the integrator output voltage either ramps up or ramps down (reset of the membrane potential). This consequently leads to a pulse train at the output of the Schmitt trigger. The operation of the circuit is further discussed below.

Assuming the neuron is starting in a resting state with $V_{mem} = 0$, the SPDT switch connects the memristor R_{mem} to OP1 and charges V_{mem}. Once $V_{mem} < V_{th,lo}$, the output of OP2, *Q*, swings high similar to the upstroke of the action potential in a biological spiking neuron. This changes the state of the SPDT switch and starts the discharge process of the capacitor C by connecting it to R_{ref} which can be thought to mimic potassium channels in biology.

Separate dc reference voltage levels V_{ref1} and V_{ref2} have been applied to the other end of the memristor and the reference resistor respectively, mimicking reversal potentials of biological ion-channels. The following relation has been maintained between the values of these voltages and V_{cm} for proper operation of the spiking neuron circuit:

$$V_{ref1} > V_{cm} > V_{ref2}$$

The value of R_{ref} has been set to be quite small so that the discharging duration (say, denoted by T_{on}) is quite low in comparison to the time required for charging (say, denoted by T_{off}), in general. Further, the difference between the voltages V_{ref1} and V_{cm} should be kept small so that the potential difference does not lead to any appreciable change in resistance of the memristive device.

An example of measured waveforms from the circuit using R_{mem} = 10kΩ is shown in Supplementary Figure 10. The output pulses have a constant width (equal to T_{on} of the integrator) similar to spikes fired from a neuron.

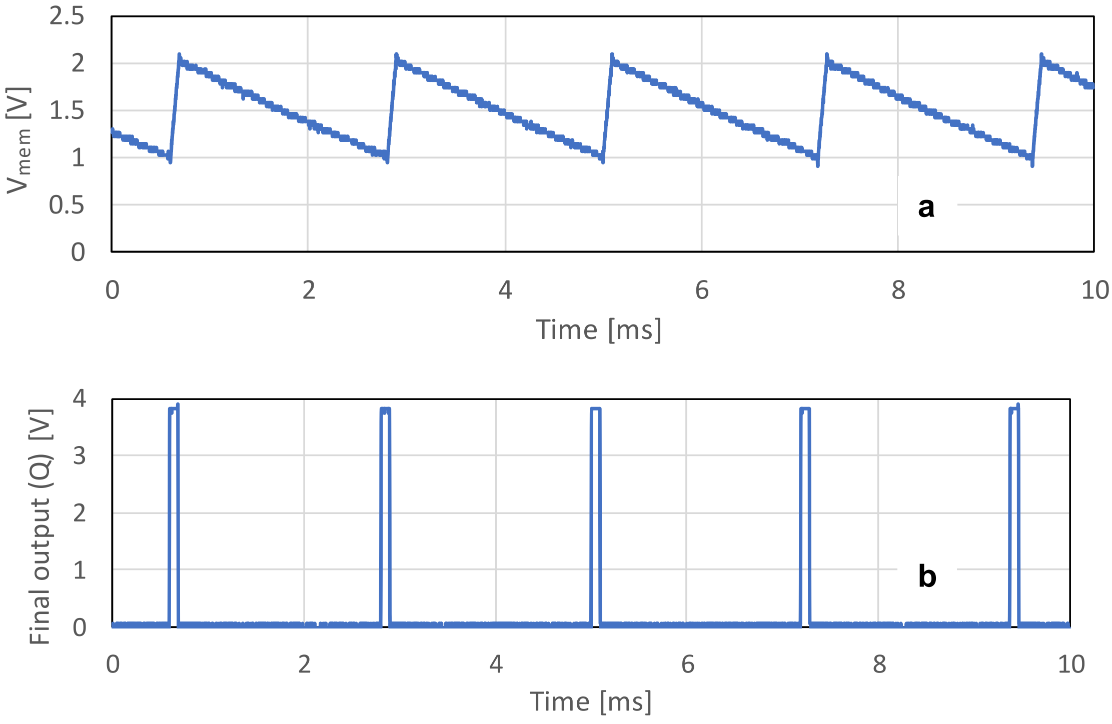

Supplementary Figure 10. Satellite Spiking Neuron (SSN) Circuit with a standard resistor. Output waveforms from the **a** integrator and **b** the comparator of the neuron circuit corresponding to a standard 10kΩ resistor used in place of R_{mem}.

Expressions of T_{on} and T_{off} durations of the neuron are given by:

$$T_{off} = \frac{R_{mem} \cdot C \cdot \Delta V_o}{V_{ref1} - V_{cm}} \quad \ldots\ldots\ldots\ldots\ldots\ldots\ldots 1$$

$$T_{on} = \frac{R_{ref} \cdot C \cdot \Delta V_o}{V_{cm} - V_{ref2}} \quad \ldots\ldots\ldots\ldots\ldots\ldots\ldots 2$$

$$where, \quad \Delta V_o = V_{th,up} - V_{th,l} \quad \ldots\ldots\ldots\ldots\ldots\ldots\ldots 3$$

The output waveform (Q) consists of pulses (of fixed width T_{on}) that repeat at a frequency (f)

given by the following expression (assuming $T_{off} >> T_{on}$):

$$f \approx \frac{1}{T_{off}} = \frac{V_{ref1} - V_{cm}}{R_{mem} \cdot C \cdot \Delta V_o} \quad \ldots\ldots\ldots\ldots\ldots\ldots.4$$

Thus, the repetition frequency is inversely proportional to the memristance, or in other words directly proportional to the charging current. By measuring this frequency or T_{off}, the resistance of the memristor can be determined as given in the expression below.

$$R_{mem} = \frac{(V_{ref1} - V_{cm}) \cdot T_{off}}{C \cdot \Delta V_o} \quad \ldots\ldots\ldots\ldots\ldots\ldots.5$$

Supplementary Table 2 presents the values of the different components and dc voltage levels as utilized in the designed circuit with discrete components.

Supplementary Table 2. Values of various components and dc reference levels as used in the experiment.

Component/Voltage	Value	Unit
C	100	nF
R_{ref}	100	Ω
R_1	100	KΩ
R_2	100	KΩ
V_{dd}	5	V
V_{cm}	2.5	V
V_{ref1}	3	V
V_{ref2}	1.65	V
V_{ref3}	2.5	V
$V_{th,up}$	2	V
$V_{th,lo}$	1	V
V_G	3	V

Next, a SWARM with a nominal high resistance state is connected to the spiking neuron. On detecting the presence of a noxious signal that is strong enough to induce a non-volatile weight update, the device resistance reduces to a low value. Measured output waveforms corresponding to high and low resistive states of the device are shown in Supplementary Figure 11. Similar to biology, the artificial PNS neuron responds to a noxious signal by an increased firing rate. For the higher memristive state, the negative pulse width (T_{off}) and frequency are measured as 0.102 s and 9.8 Hz respectively. On the other hand, for the lower resistive state, the negative pulse width and frequency are found to be 45.4 μs and 7.39 kHz respectively.

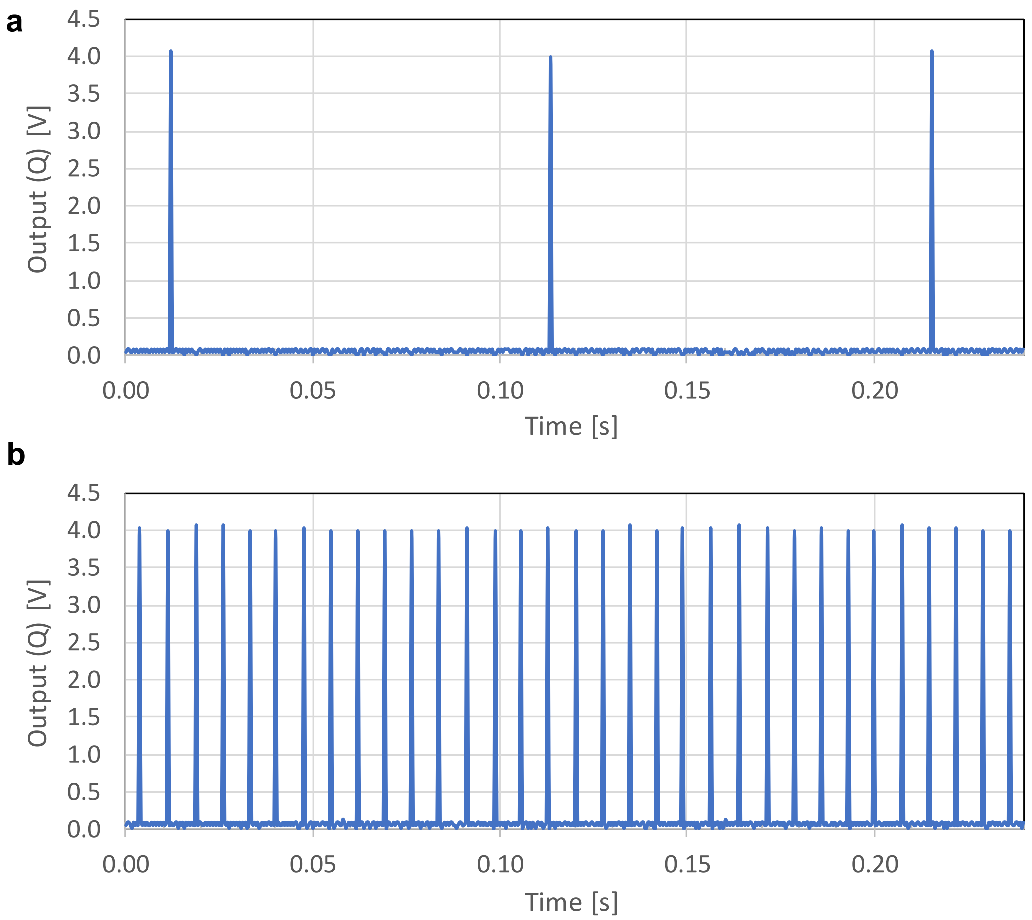

Supplementary Figure 11. Satellite Spiking Neuron (SSN) Circuit with a Satellite Weight Adjusting Resistive Memory (SWARM) resistor. Spiking waveforms corresponding to **a** the high resistive and **b** low resistive states of a SWARM that associates to absence and presence of a noxious signal.

Supplementary Note 6. Self-Healing Ionic Dielectrics and Neuromorphic Memtransistors

The self-healing nature of the ion gels is investigated via a combination of Fourier transform infrared (FTIR) spectroscopy, differential scanning calorimetry (DSC) and thermogravimetric analysis (TGA) as explained below.

The ionic liquid inclusions form electrochemically and thermally stable ion-dipole interactions with the polar polymer host, inducing a plasticizing effect. These molecules increase the free volume between the polymer chains, allowing for greater chain mobility, indicated by the lowering of glass transition temperatures (T_g). The increased flowability enhances mechanical flexibility as well as vastly reduces the healing temperatures[17]. Fourier transform infrared (FTIR) spectroscopy is used to investigate interactions between the polymer and ionic liquid in the ion gel by monitoring vibrational energies of the chemical bonds (Supplementary Figure 12).

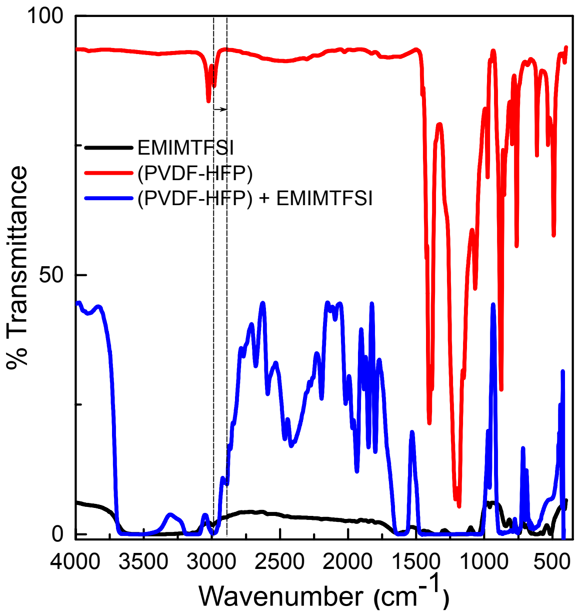

Supplementary Figure 12. Fourier transform infrared (FTIR) spectroscopy. FTIR spectra of the (PVDF-HFP) polymer, Ionic liquid (EMITFSI) and ((PVDF-HFP) +EMITFSI) ion gel.

Addition of EMIMTFSI into the polymer matrix result in the appearance of new peaks, namely: in-plane C-H bending peak of imidazolium ring at 846 cm^{-1}, C-S and S-N stretching peak at 788 cm^{-1}, S-N-S asymmetric stretching peak at 1054 cm^{-1}, N-H stretching peak at 1226 cm^{-1} and SO_2 asymmetric stretching peak of the counter anion TFSI at 1349 cm^{-1}. It was previously reported that addition of EMIMTFSI into the P(VDF-HFP) matrix resulted in shifting of antisymmetric stretching of CF_2 band to higher wavenumber[18]. This observation was attributed to strong ion–dipole interactions between the polymer and the imidazolium-based ionic liquid. In our case, such putative interactions are also found to affect the stretching vibration of the hydrocarbon chain within the P(VDF-HFP). In particular, a notable shift to lower wavenumber was observed in its C-H stretching peak from ca. 2938 cm^{-1} to ca. 2874 cm^{-1} (Supplementary Figure 12)[19]. As wavenumber correlates proportionally with the strength of the bond formed between the participating atoms[20], this suggests that interactions between the polymer and the ionic liquid also lead to weakening of the bonding in the latter's functionality.

Differential scanning calorimetry (DSC) results provide more direct evidence on the plasticizing effect. As shown in Supplementary Figure 13, the glass transition temperature of the material is lowered from −14.5 to −45.5 °C upon the addition of 33wt% EMIMTFSI. The ion–dipole interaction and the plasticizing effect both contribute to the self-healing property of the materials. Thermogravimetric analysis (TGA) reveals thermal stability of the ion gel above 350 °C (Supplementary Figure 14).

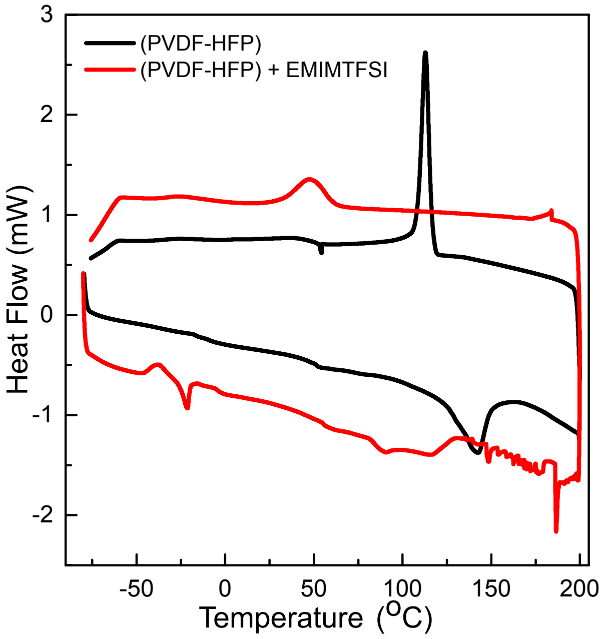

Supplementary Figure 13. Differential scanning calorimetry (DSC). DSC spectra of the (PVDF-HFP) polymer, Ionic liquid (EMITFSI) and ((PVDF-HFP) +EMITFSI) ion gel.

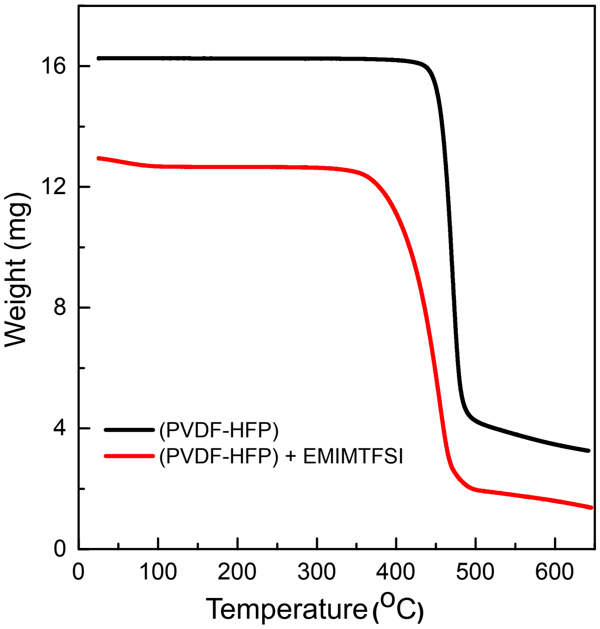

Supplementary Figure 14. Thermogravimetric analysis (TGA). TGA spectra of the (PVDF-HFP) polymer, Ionic liquid (EMITFSI) and ((PVDF-HFP) +EMITFSI) ion gel.

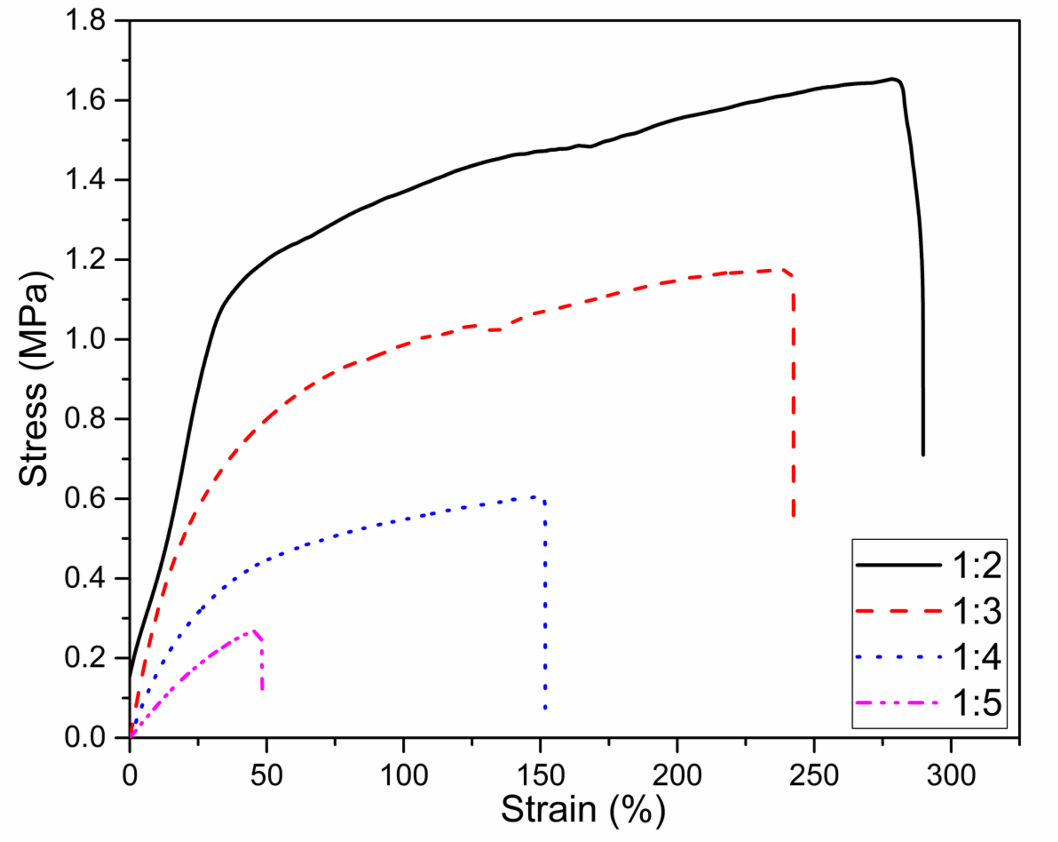

Supplementary Figure 15. Mechanical Characterization. Stress strain curves for different loadings of EMITFSI in PVDF-HFP: EMITFSI ionic gel. Increasing concentration of the ionic liquid leads to softening and reduction in Young's modulus of the gel but is accompanied with reduced stretchability of the matrix. 1:4 (PVDF-HFP: EMITFSI) shows a Young's modulus of 2 MPa and an ultimate strain of 150%; and is chosen for further investigations.

Mechanical properties of these films as a function of the weight ratio of the ionic liquid are investigated by uniaxial tensile tests. As seen in Supplementary Figure 15, increasing the content of EMITFSI in the ionic gel leads to distinct stress-strain curves emphasizing the tuning of mechanical properties that can be achieved by varying the concentration of ionic liquid in the gel. Softening of the matrix and decrease in the Young's modulus of the ionic gels with higher EMITFSI content is evident from the stress-strain curves. However, it is important to mention here that the stretchability of the material reduces with increasing content of EMITFSI, evident from the observed reduction of ultimate strain (maximum strain at break). At 1:4 (PVDF-HFP:EMITFSI), the material exhibits a Young's modulus of 2 MPa, comparable to silicones, a typical elastomeric material utilized in the field of stretchable and flexible electronics[21,22]. The measured Young's modulus at 1:5 ((PVDF-HFP:EMITFSI) is 1MPa compared to 4 MPa at 1:2 ((PVDF-HFP:EMITFSI). These measured moduli of mechanical

stiffness lie in the range of biological materials[23]. As mentioned earlier, there is a reduction in the ultimate strain with increasing content of ionic liquid, with a measured ultimate strain of 285 % for 1:2 reducing to 150 % for 1:4 and only around 50 % for 1:5. Based on the combination of measured mechanical parameters; mechanical softness, Young's modulus and stretchability; 1:4 (PVDF-HFP: EMITFSI) is chosen for device fabrication.

Healing of the mechanical integrity of the ionic gel matrix is analysed by monitoring the stress-strain curve. Supplementary Figure 16 shows the stress-strain curve for PVDF-HFP: EMITFSI (1:4) samples, for as fabricated sample (pristine) and healed sample; healing done for a completely cut sample at room temperature (RT) for 24 hours. It is worthwhile to mention that the healed sample has similar slope for the stress-strain curve as the pristine sample, indicating no change in the Young's modulus and mechanical stiffness of the material during the healing process. The sample shows a healing efficiency of 27 % in terms of maximum strain at break (ultimate strain) and an impressive 67 % in terms of peak load. It is evident from the photographs in Figure-S16 that the breakage of the healed sample originates from the place where the sample was initially cut and healed. It can be implied that the healing at room temperature for 24 hours does not completely heal back the sample and may require longer time duration required for complete recovery of mechanical properties. The healing process may also be expedited by application of some external stimulus like heat to expedite the complete recovery[18,24].

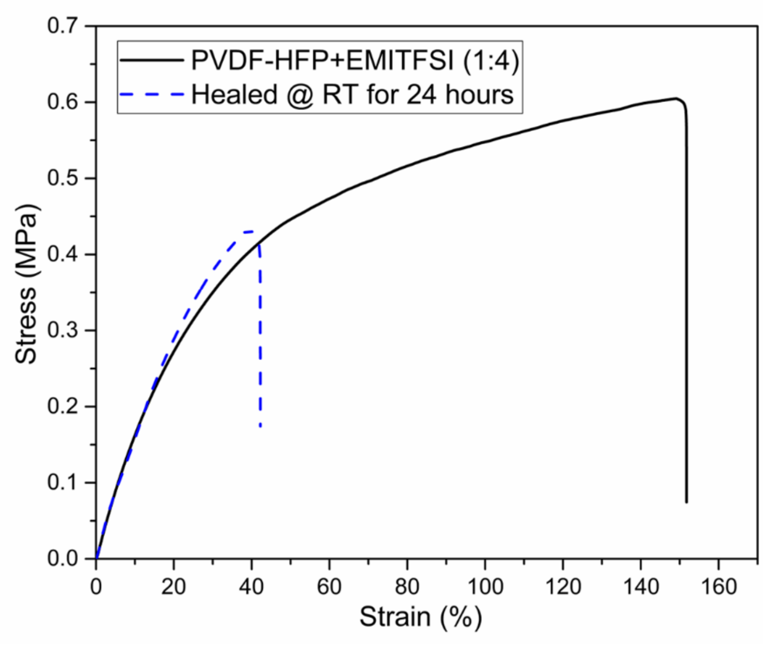

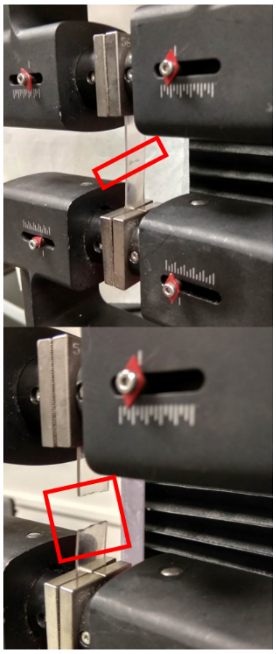

Supplementary Figure 16. Healing of the mechanical integrity. Stress strain curves for the pristine and healed sample (PVDF-HFP: EMITFSI (1:4)). Healing is done at room temperature (RT) for 24 hours by keeping the

samples in contact. There are visible signs of the healed cut on the sample after the RT healing for 24 hours. Under uniaxial stress, the healed sample tears off, from the same location where the sample was cut and healed.

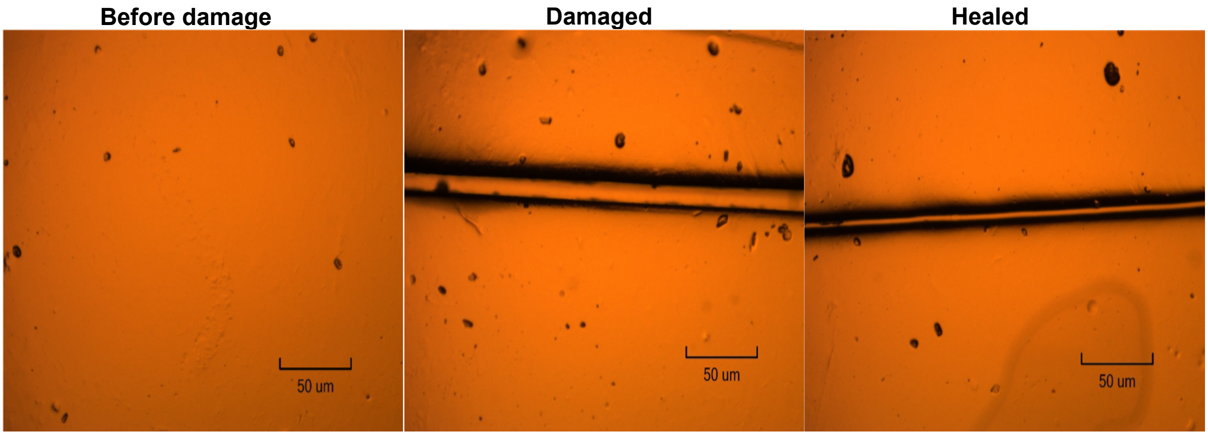

Supplementary Figure 17. Optical microscope images of the ion gel under various stages of damage and heal process. Upon injury, the ionic liquid inclusions trigger the healing process by improving the thermal mobility of the polymer housing via a plasticizing mechanism. The optical microscope images are shown in this figure and the corresponding SEM images are shown in main text Fig. 4b.

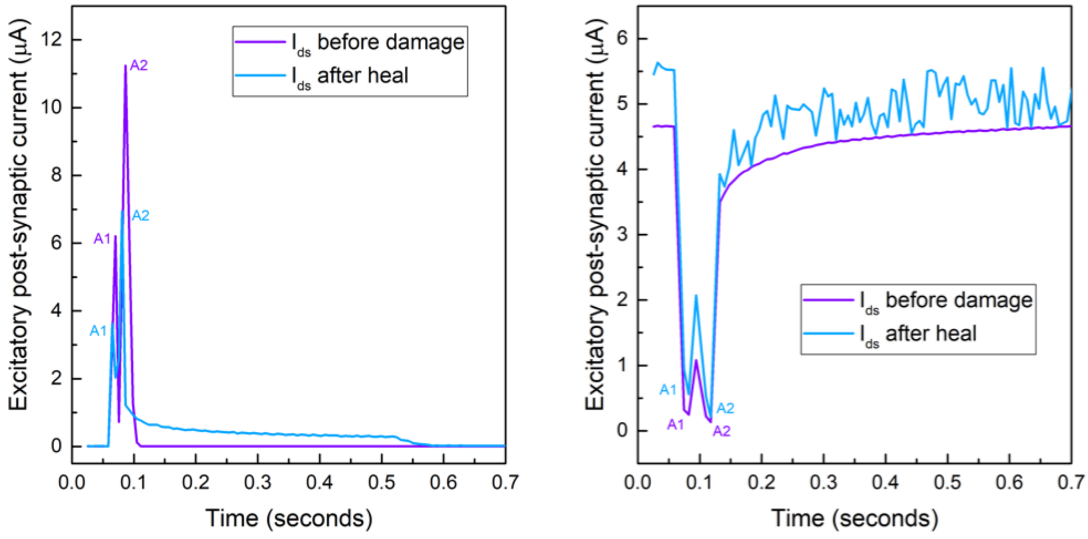

Supplementary Figure 18. Short-term Plasticity in Satellite Weight Adjusting Resistive Memories (SWARMs). A pair of presynaptic action potentials (+1.5V, pulse width = 20 ms, interval = 10 ms) triggers a pair of excitatory postsynaptic currents (EPSCs) with increasing amplitude. This phenomenon known as paired-pulse facilitation (PPF) reflects the number of residual carriers during ion migration relaxation kinetics in the ionic-gated mode (left). Reversal of polarity of the presynaptic action potentials (-1.5 V) result in paired-pulse depression (PPD) with the indices dependent on pulse width and interval of the presynaptic action potentials (right), similar to facilitation. The figure shows representative plots of the drain current before damage and after the healing process for both PPF and PPD.

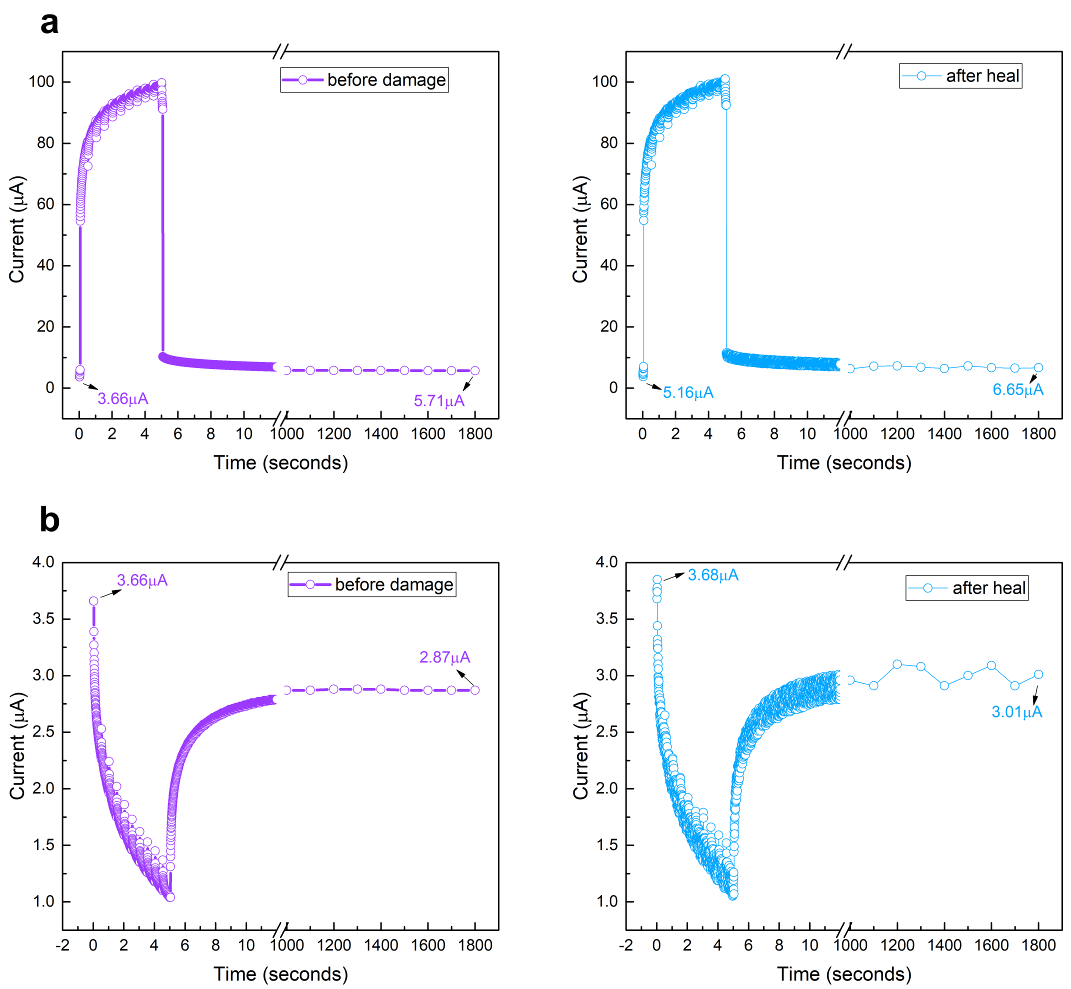

Supplementary Figure 19. Long-term Plasticity in Satellite Weight Adjusting Resistive Memories (SWARMs). Persistent biasing of the gate voltage (please refer to the input waveforms shown in Supplementary Figure 8) results in long-term potentiation (LTP) and depression (LTD), widely considered a primary mechanism for learning and memory. Representative plots of the drain current before damage and after the healing process for **a** LTP and **b** LTD.

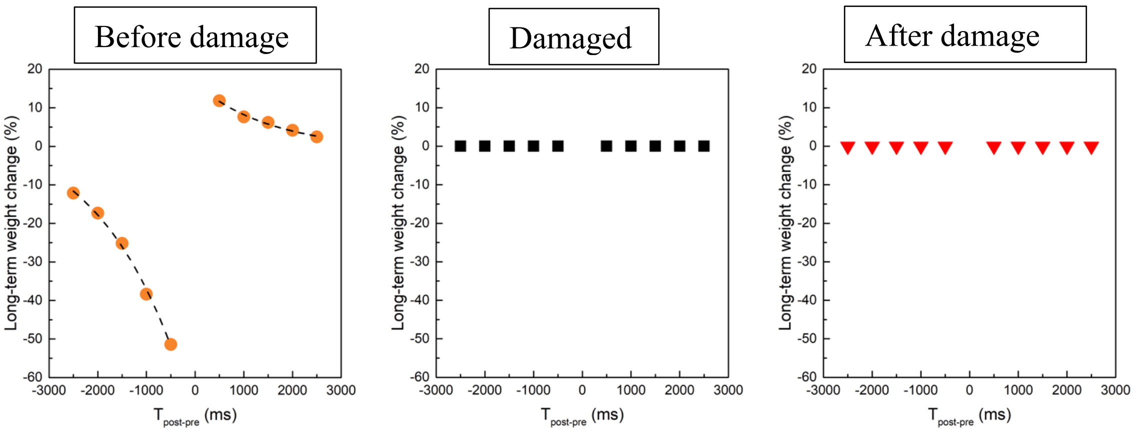

Supplementary Figure 20. Vulnerability of SiO_2 dielectric to mechanical damage. The figure shows comprehensive electrical characterization of artificial synaptic transistors configured in the bottom SiO_2-gated

mode at various stages of the damage and recovery process. The inability of these devices to repair themselves upon injury reflects the vulnerability of conventional dielectrics like SiO_2 to unexpected mechanical damage.

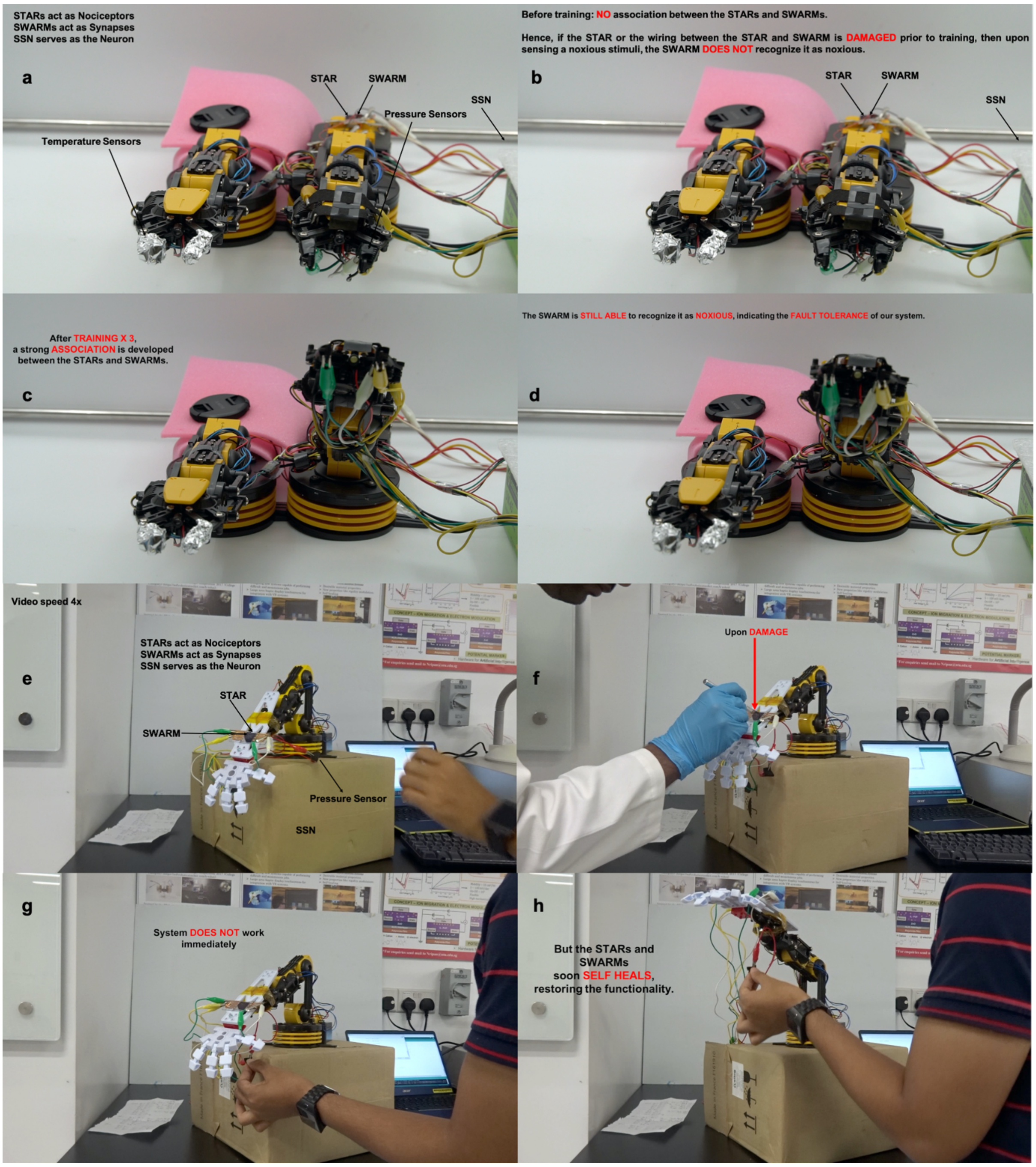

Supplementary Figure 21. Decentralizing intelligence with memristive neuromorphic elements. a Satellite Threshold Adjusting Receptors (STARs) act as nociceptors, Satellite Weight Adjusting Resistive Memories (SWARMs) as synapses and satellite spiking neurons (SSNs) as neurons. **b** If the system is not trained, no association is developed between the STARs and SWARMs and hence, noxious signals cannot not be detected upon damage to the STAR or interconnection between the STARs and SWARMs. **c** However, once the system is trained to detect noxious stimuli through the 3-step learning process comprising of threshold detection (STARs), associative learning (SWARMs) and integration (SSNs), noxious signals can still be detected and learnt even after damage to the STAR or interconnection between the STARs and SWARMs **d**. **e-h** Functional recovery of the

system upon mechanical damage. **e** Experimental setup. **f-g** If the ion gel of the STARs and SWARMs is damaged, the system does not work. **h** However, the STARs and SWARMs soon self-heals restoring the circuit functionality. Please refer to Supplementary Movies 1-3 for more details.

Supplementary References

1. Lee, K. H. *et al.* "Cut and stick" rubbery ion gels as high capacitance gate dielectrics. *Adv. Mater.* **24**, 4457–4462 (2012).
2. Zhu, L. Q., Wan, C. J., Guo, L. Q., Shi, Y. & Wan, Q. Artificial synapse network on inorganic proton conductor for neuromorphic systems. *Nat. Commun.* **5**, 3158 (2014).
3. Yoon, J. H. *et al.* An artificial nociceptor based on a diffusive memristor. *Nat. Commun.* **9**, 417 (2018).
4. Kulkarni, M. R. *et al.* Field-Driven Athermal Activation of Amorphous Metal Oxide Semiconductors for Flexible Programmable Logic Circuits and Neuromorphic Electronics. *Small* **15**, 1901457 (2019).
5. Ueno, K. *et al.* Discovery of superconductivity in KTaO 3 by electrostatic carrier doping. *Nat. Nanotechnol.* **6**, 408 (2011).
6. Gallagher, P. *et al.* A high-mobility electronic system at an electrolyte-gated oxide surface. *Nat. Commun.* **6**, 6437 (2015).
7. Ye, J. T. *et al.* Liquid-gated interface superconductivity on an atomically flat film. *Nat. Mater.* **9**, 125–128 (2010).
8. Jeong, J. *et al.* Giant reversible, facet-dependent, structural changes in a correlated-electron insulator induced by ionic liquid gating. *Proc. Natl. Acad. Sci.* **112**, 1013 LP – 1018 (2015).
9. Wang, Y. *et al.* Structural phase transition in monolayer MoTe 2 driven by electrostatic doping. *Nature* **550**, 487 (2017).
10. Lu, N. *et al.* Electric-field control of tri-state phase transformation with a selective dual-ion switch. *Nature* **546**, 124 (2017).
11. John, R. A. *et al.* Low-Temperature Chemical Transformations for High-Performance Solution-Processed Oxide Transistors. *Chem. Mater.* **28**, 8305–8313 (2016).
12. Socratous, J. *et al.* Electronic Structure of Low-Temperature Solution-Processed Amorphous Metal Oxide Semiconductors for Thin-Film Transistor Applications. *Adv. Funct. Mater.* **25**, 1873–1885 (2015).
13. Li, Y. *et al.* Ultrafast synaptic events in a chalcogenide memristor. *Sci. Rep.* **3**, (2013).
14. D'Souza, P., Liu, S.-C. & Hahnloser, R. H. R. Perceptron learning rule derived from

spike-frequency adaptation and spike-time-dependent plasticity. *Proc. Natl. Acad. Sci.* **107**, 4722–4727 (2010).

15. Song, S., Miller, K. D. & Abbott, L. F. Competitive Hebbian learning through spike-timing-dependent synaptic plasticity. *Nat. Neurosci.* **3**, 919–926 (2000).
16. Caporale, N. & Dan, Y. Spike timing–dependent plasticity: a Hebbian learning rule. *Annu. Rev. Neurosci.* **31**, 25–46 (2008).
17. Tiwari, N., Ho, F. & Mathews, N. A rapid low temperature self-healable polymeric composite for flexible electronic devices. *J. Mater. Chem. A* **6**, 21428–21434 (2018).
18. Cao, Y. *et al.* A transparent, self-healing, highly stretchable ionic conductor. *Adv. Mater.* **29**, 1605099 (2017).
19. Takamuku, T. *et al.* Microscopic interactions of the imidazolium-based ionic liquid with molecular liquids depending on their electron-donicity. *Phys. Chem. Chem. Phys.* **16**, 23627–23638 (2014).
20. Atkins, P. & Paula, J. Molecular spectroscopy 1: rotational and vibrational spectra. *Atkins' Phys. Chem.* **8**, 430–480 (2006).
21. McDonald, J. C. & Whitesides, G. M. Poly(dimethylsiloxane) as a Material for Fabricating Microfluidic Devices. *Acc. Chem. Res.* **35**, 491–499 (2002).
22. Tiwari, N., Rajput, M., Chien, N. A. & Mathews, N. Highly Transparent and Integrable Surface Texture Change Device for Localized Tactile Feedback. *Small* **14**, 1702312 (2018).
23. Rus, D. & Tolley, M. T. Design, fabrication and control of soft robots. *Nature* **521**, 467 (2015).
24. Tiwari, N., Rajput, M., Kulkarni, M. R., John, R. A. & Mathews, N. Healable and flexible transparent heaters. *Nanoscale* **9**, 14990–14997 (2017).
