# Supplementary material for: Self healable neuromorphic memtransistor elements for decentralized sensory signal processing in robotics
Source: Nat Commun. 2020 Aug 12;11:4030. doi: 10.1038/s41467-020-17870-6 (PMC7424569; doi:10.1038/s41467-020-17870-6)
Supplement: Supplementary file 2 — Description of Additional Supplementary Files [file 41467_2020_17870_MOESM2_ESM.pdf]

Title: Supplementary Movie 1:
Description: Associative Learning enables fault tolerance

Title: Supplementary Movie 2:
Description: Self-healable Memristive Neuromorphic Elements

Title: Supplementary Movie 3:
Description: Fusing multiple sensory inputs
